# Integrin signalling regulates the expansion of neuroepithelial progenitors and neurogenesis via Wnt7a and Decorin

**DOI:** 10.1038/ncomms10354

**Published:** 2016-02-03

**Authors:** K. Long, L. Moss, L. Laursen, L. Boulter, C. ffrench-Constant

**Affiliations:** 1 MRC Centre for Regenerative Medicine, University of Edinburgh, Edinburgh EH16 4UU, UK; 2 Department of Molecular biology and Genetics, Aarhus University, Gustav Wieds Vej 10 C, 8000 Aarhus C, Denmark; 3 MRC Human Genetics Unit, Institute for Genetics and Molecular Medicine, Crewe Road, Edinburgh EH4 2XU, UK

## Abstract

Development of the cerebral cortex requires regulation of proliferation and differentiation of neural stem cells and a diverse range of progenitors. Recent work suggests a role for extracellular matrix (ECM) and the major family of ECM receptors, the integrins. Here we show that enhancing integrin beta-1 signalling, by expressing a constitutively active integrin beta-1 (CA*β1) in the embryonic chick mesencephalon, enhances neurogenesis and increases the number of mitotic cells dividing away from the ventricular surface, analogous to sub-apical progenitors in mouse. Only non-integrin-expressing neighbouring cells (lacking CA*β1) contributed to the increased neurogenesis. Transcriptome analysis reveals upregulation of Wnt7a within the CA*β1 cells and upregulation of the ECM protein Decorin in the neighbouring non-expressing cells. Experiments using inhibitors in explant models and genetic knock-downs *in vivo* reveal an integrin-Wnt7a-Decorin pathway that promotes proliferation and differentiation of neuroepithelial cells, and identify Decorin as a novel neurogenic factor in the central nervous system.

## Abstract (web summary)

The extracellular matrix is suggested to play a role in neurogenesis, but it is unclear what role integrin signalling may play in the developing neuroepithelium. Here, in chick, Long *et al*. show that expression of constitutively active integrin beta-1 enhances neurogenesis via a novel Wnt7 and decorin pathway.

## Graphical abstract

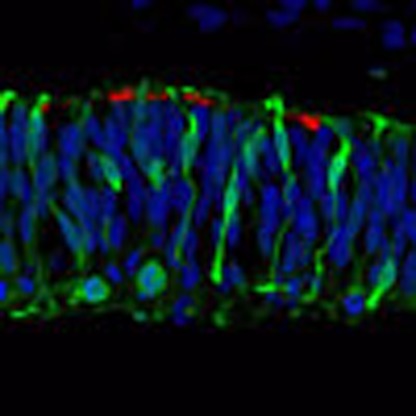

The development of the central nervous system (CNS) requires the formation of billions of neurons from a population of proliferating neural stem cells (NSCs). Initially, NSCs undergo rounds of proliferative divisions, expanding the progenitor pool, before switching to neurogenic or asymmetric divisions, to generate amplifying progenitor cells and/or post-mitotic neurons. The differences in the regulation of proliferation verses differentiation and the expansion of progenitors enables the expansion of neurogenesis seen in mammalian brains.

A number of signals that regulate the balance between stem cell maintenance and differentiation have been defined, including the Notch, fibroblast growth factor, Sonic Hedgehog, Bone morphogenetic protein and Wnt pathways^1^,^2^,^3^,^4^,^5^,^6^,^7^,^8^,^9^,^10^,^11^,^12^. Our knowledge of these signals, however, remains incomplete, and the characterization of the complete repertoire is important both for understanding the mechanisms of developmental abnormalities of the cortex and also for devising strategies to generate neurons *ex vivo* for disease modelling and drug screening.

A role for extracellular matrix (ECM) in neurogenesis is suggested by the high expression of ECM proteins, such as laminin, in both the pial basement membrane overlying the developing neuroepithelium and in the ventricular zone (VZ), the area immediately adjacent to the ventricular surface where the majority of NSCs divide^13^,^14^. ECM signalling was also found to be a predominant feature in gene expression analysis of regions of the developing CNS where neural precursors undergo both self-renewing and neurogenic divisions in both the mouse and human CNS^15^.

This expression of ECM in the VZ raises the question as to the role of integrins, the principal ECM receptors, in the regulation of NSC and precursor behaviour. Perturbation studies to address this question using genetic knockout strategies, disintegrin molecules, or the injection of blocking antibodies into the ventricle, have led to detachment of basal and apical NSC processes, respectively, from the pial basement membrane or the ventricular surface, often resulting in apoptosis of these cells^14^,^16^,^17^,^18^,^19^. A direct role in signalling, in addition to the adhesive function shown by these perturbation experiments, is suggested by two sets of experiments examining amplifying progenitor populations. First, those using integrin activating antibodies leading to an expansion and cell cycle re-entry of the intermediate progenitors in the mouse sub-VZ (SVZ). Second, those in which functional disruption of integrins decreased the population of proliferating basal progenitors (BPs) in ferret and human, suggesting a role in the expansion of outer-sub-VZ (OSVZ) progenitors^18^,^20^.

Although these studies suggest an important contribution for integrin/ECM signalling in both adhesion and signalling during neurogenesis and the proliferation of progenitors, establishing the exact role of integrins within the repertoire of signals regulating neurogenesis requires that we define the cellular and molecular consequences of integrin signalling in NSCs. The approaches used above have significant drawbacks as experimental strategies to address these questions of mechanism; blocking antibodies lead to the secondary effects of architectural disruption created by the loss of adhesion, while activating antibodies target all cells equally and so do not allow the distinction between cell autonomous and non-autonomous effects.

In this study, we have therefore expressed a constitutively active integrin beta-1 (CA*β1) within the neuroepithelium, with the logic being that this gain-of-function mutation allows the examination of integrin function without the loss of adhesion observed in our previous blocking-antibody experiments. We have used electroporation in the early chick mesencephalon as an experimental model to examine both cell autonomous and non-autonomous effects of increased integrin signalling in an accessible and very simple neuroepithelium; the multiple progenitor types seen in mammalian neocortices are rarely formed, simplifying the cell biology. Our results confirm recent work showing that integrin signalling can expand the neuroepithelium and reveal a novel pathway by which integrin signalling promotes this expansion, with neurogenesis enhanced by the secretion of Wnt7a and the upregulation of the ECM link protein Decorin.

## Results

### Manipulation of itgβ1 within the chick neuroepithelium

Integrin β1 (itgβ1) has previously been shown to be expressed on both NSCs and the embryonic neuroepithelium of the mouse and human^13^,^15^,^16^,^18^,^21^. We confirmed that itgβ1 is expressed within the mesencephalic neuroepithelium of the chick embryo during the early stages of neurogenesis (E2-4) via immunohistochemistry (Supplementary Fig 1). We also confirmed that integrin alpha 6 (itgα6), which dimerizes with itgβ1 to form the laminin receptor, was expressed within the chick neuroepithelium in a similar pattern (Supplementary Fig 1), suggesting the presence of functional itgα6β1 expression in the chick as in the mouse.

We used three human itgβ1 constructs to manipulate signalling levels in the neuroepithelium: a wild-type itgβ1 (hWTβ1), an itgβ1 lacking the intracellular domain (ECβ1) and a CA*β1 (refs 22, 23) (Supplementary Table 1). The functional expression of these constructs was first confirmed using a chick fibroblast cell line (DF-1). A human itgβ1-specific antibody was used to distinguish between the electroporated and endogenous chick itgβ1. All itgβ1 constructs were detected on the cell surface of live DF-1 cells via fluorescence-activated cell sorting (FACS; Supplementary Fig 1).

The predicted function of these constructs was confirmed by assessing activation (phosphorylation) of a well-known downstream target of itgβ1, focal adhesion kinase (FAK). Immunohistochemical detection of pFAK^Y397^ levels showed an increase in FAK activity upon expression of CA*β1 in DF-1 cells, whereas the other constructs had no effect (Supplementary Fig 1), confirming the activity of the construct within chick cells.

We next confirmed the co-expression of the integrin constructs *in ovo* with the co-electroporated cytoplasmic green fluorescent protein (GFP), used to mark the electroporated area. Strong co-expression of the GFP and itgβ1 constructs was detected by immunohistochemistry at E4 (HH23), 48 h after electroporation (Supplementary Fig 1), as was increased pFAK^Y397^ levels upon expression of CA*β1 within the neuroepithelium (Supplementary Fig 1). By electroporation of activating integrin constructs, we created a mosaic population of targeted and non-targeted NSCs, allowing the investigation of integrin signalling both within (cell autonomous) and between cells (non-cell autonomous).

### Expression of CA*β1 expands the neuroepithelium

**Figure 1:**
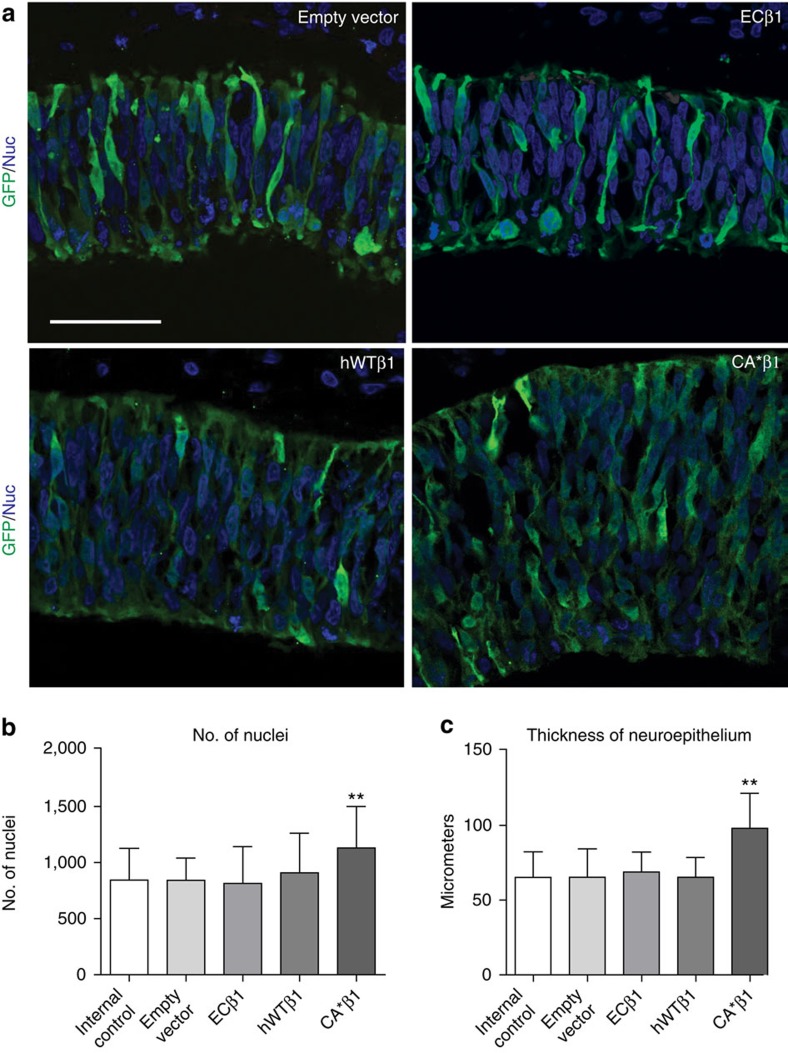
Expression of CA*β1 expands the neuroepithelium. (**a**) Immunostaining for GFP in E4 midbrain neuroepithelium electroporated with the empty vector, wild-type integrin β1 (itgβ1, hWTβ1), itgβ1 lacking the intracellular domain (ECβ1) or constitutively active integrin beta-1 (CA*β1). Scale bar, 20 μm. (**b**) Quantification of the number of nuclei per field. *n*>19. (**c**) Quantification of the thickness of the neuroepithelium. *n*>13. All graphs: mean and s.d. **P<0.01, one-way analysis of variance.

Expression of CA*β1 within the early midbrain at E2, the onset of neurogenesis, resulted in the radial expansion (that is, thickening) of the neuroepithelium 48 h later at E4 (Fig. 1a). The number of nuclei per radial field was increased, as was the radial thickness of the neuroepithelium (Fig. 1b,c). Less marked changes were observed by E3, 24 h after electroporation, but did not reach significance at that early time point (Supplementary Fig 2). The lateral length of the neuroepithelium was not affected, as evidenced by the lack of folding at either the ventricular or pial surface, suggesting expression of CA*β1 promotes a radial and not lateral expansion of the early midbrain neuroepithelium. Expression of the empty vector control, ECβ1 or hWTβ1 had no effect on the number of nuclei per field or the thickness of the neuroepithelium at either E3 or E4 (Fig. 1a–c and Supplementary Fig 2).

**Figure 2:**
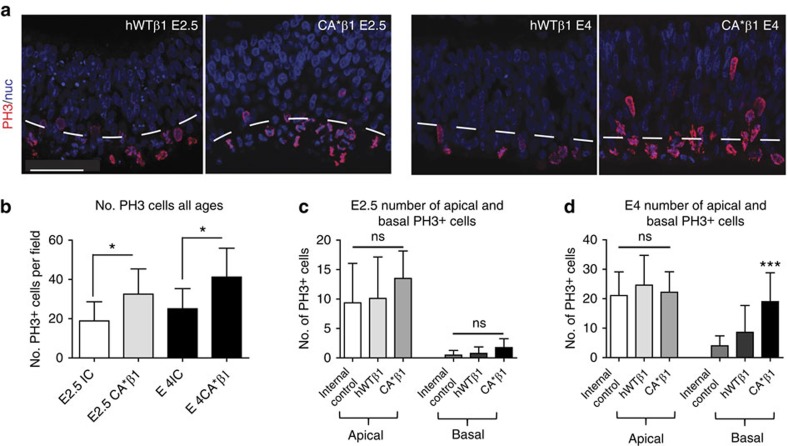
Expression of CA*β1 expands the progenitor pool and increases sub-apical divisions. (**a**) Immunostaining for PH3 in E2.5 and E4 midbrain neuroepithelium electroporated with hWTβ1 or CA*β1. Dashed white line labels the apical area defined as two or three cell diameters above the ventricular surface. Note the increase in dividing cells above (basal to) the dotted line in the CA*β1-expressing neuroepithelium. Scale bar, 20 μm. (**b**) Quantification of the number of PH3^+^ cells. *n*>4. (**c**) Quantification of the location of PH3^+^ cells at E2.5. *n*>5. (**d**) Quantification of the location of PH3^+^ cells at E4. *n*>5. Scale bar, 20 μm. All graphs: mean and s.d., *P<0.05, ***P<0.001, one-way analysis of variance.

To determine the mechanism for the expanded neuroepithelium resulting from expression of CA*β1, we quantified rates of cell death and cell proliferation. No change in cell death was observed with TUNEL staining across all conditions (Supplementary Fig 3), suggesting that the increase in cell number is not due to a decrease in cell death. When we examined changes in proliferation using Phosphohistone H3 (PH3), a marker of late G2/M phase, the number of PH3^+^ cells was significantly increased at E2.5, 12 h after electroporation, and at E4, 48 h after electroporation, when CA*β1 was expressed (Fig. 2a,b). Interestingly, at E2.5, the majority of PH3^+^ cells are located apically at the ventricular surface, but at E4 a significant proportion of PH3^+^ cells are located sub-apically (more than two or three cell diameters above the ventricular surface; Fig. 2a,c,d).

To exclude the possibility that the appearance of these sub-apically proliferating cells resulted from disruption of the normal architecture of the neuroepithelium, we performed four sets of experiments. First, we examined the expression of the apical complex markers, Par3, aPKC or N-cadherin, and the distribution of actin as revealed by phalloidin, to study the process attachment of neuroepithelial cells to the ventricular surface. Neither the expression of apical complex proteins or the distribution of phalloidin binding was affected by the expression of CA*β1, showing that apical process morphology was maintained (Supplementary Fig 4). Second, we counted the total and GFP^+^ number of end feet per field using en-face imaging of Phallodin labelling of the ventricular surface in whole-mount midbrains, finding that these were also not altered upon expression of CA*β1 (Supplementary Fig 4). Third, we quantified nuclear density in the apical area of the neuroepithelium, showing this was also unaffected, suggesting cells are not accumulating at the ventricular surface (Supplementary Fig 4). Last, we examined basal process adhesion by visualizing expression of the basal basement membrane components, laminin and collagen IV. These were unaffected upon expression of CA*β1, with cells evenly distributed from the ventricular to the pial surface (Supplementary Fig 4). Taken together, these results indicate the neuroepithelium has maintained normal polarity and morphology despite the activation of integrin signalling in a subpopulation of neuroepithelial cells.

**Figure 3:**
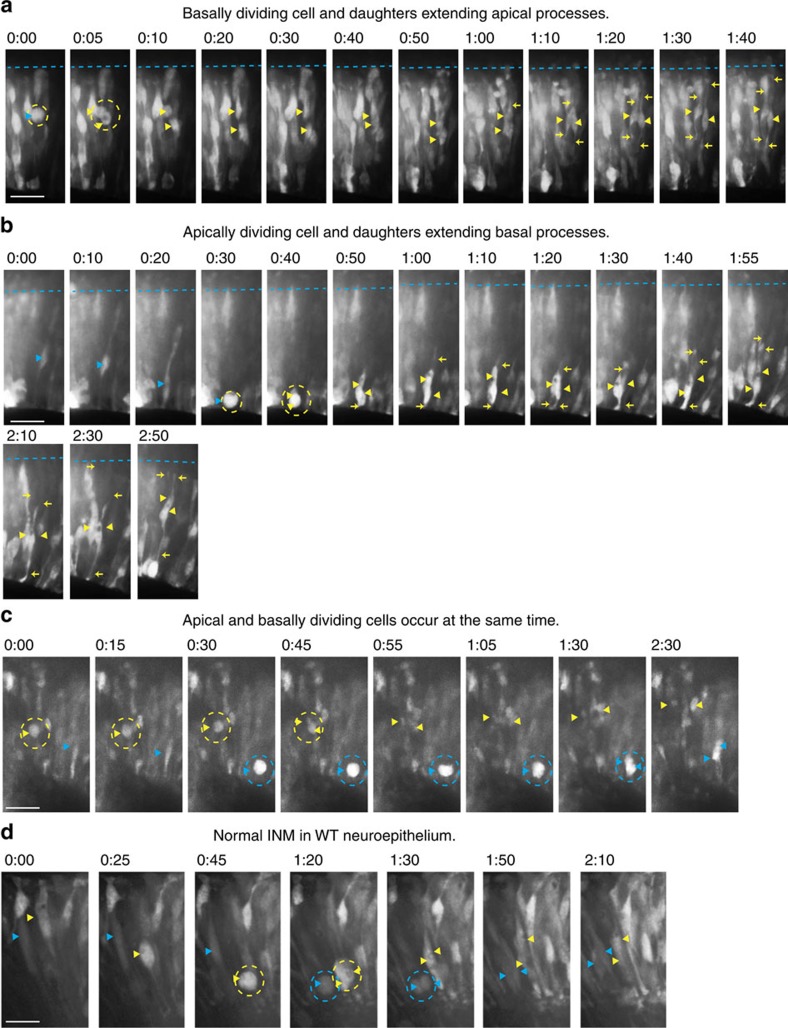
Live imaging of CA*β1-expressing neuroepithelium shows normal inter-kinetic nuclear migration and process extension alongside sub-apically dividing cells. All panels show images taken from the live time-lapse imaging of the hWTβ1- or CA*β1-expressing neuroepithelium, imaged 24–48 h after electroporation, with cells visualized using cytoplasmic GFP fluorescence. The apical surface is always at the bottom of the image, basal at the top. Filled arrowheads: cell bodies; arrows: cell processes; blue dashed line: basal surface; dashed circles: dividing cells. 0:00 (h:mm) donates the time of the first image. (**a**) CA*β1-expressing cells dividing sub-apically produce daughter cells that re-extend an apical process. (**b**) CA*β1-expressing cells dividing apically produce daughter cells that re-extend a basal process. (**c**) Apical and sub-apical divisions of CA*β1-expressing cells occur simultaneously within the neuroepithelium. (**d**) Normal inter-kinetic nuclear migration (INM) occurs within the hWTβ1-expressing neuroepithelium.Scale bars, 20 μm.

Next, therefore, we asked whether these cells were similar to sub-apical progenitors (SAPs) in the mouse—a newly described population of cells that divide within the VZ away from the ventricular surface. These cells retain apical adhesion and are thought to be the initial evolutionary step in the expansion of the neuroepithelium, enabling an increase in the number of dividing progenitors while overcoming the space constraints of the ventricular surface. We asked whether the cells in the chick have these apical properties despite their sub-apical proliferative location in two ways. First, we showed that GFP^+^ sub-apical PH3^+^ cells have apical and basal attachments (Supplementary Fig 5) and expressed the apical progenitor marker Sox2 but not the intermediate progenitor marker Tbr2 (Supplementary Fig 5). Second, we performed live imaging studies on midbrains electroporated at E2 and imaged 24–48 h later for a minimum of 16 h. The cytoplasmic GFP used to mark expression of CA*β1, or hWTβ1 in controls, allowed visualization of the entire cell, including the apical and basal processes. Confirming the lack of disruption of neuroepithelial architecture, imaging showed the morphology of the cells within the neuroepithelium was maintained, with both the apical and basal processes attached to their respective surfaces (Fig. 3a,b), and that the majority of cells in the CA*β1-expressing neuroepithelium underwent normal inter-kinetic nuclear migration (Fig. 3a,b and Supplementary Movies 1 and 2), migrating from the basal surface to the apical surface to divide, with the daughter cells extending new processes up to the basal surface before the cells migrate basally to continue the cell cycle (Fig. 3b and (Supplementary Movie 3). The sub-apically dividing cells were shown to divide away from the apical surface and when their daughter cells could be followed, we observed re-extension of their apical processes towards the apical surface (Fig. 3c and Supplementary Movie 4). These sub-apical divisions occurred simultaneously with apical divisions (Fig. 3d and Supplementary Movie 5), confirming that expression of CA*β1 generates a new population of cells dividing sub-apically and, taken together with evidence for apical process re-formation, that these are analogous to the SAPs in the mouse.

### CA*β1 increases neurogenesis in a non-cell autonomous manner

**Figure 4:**
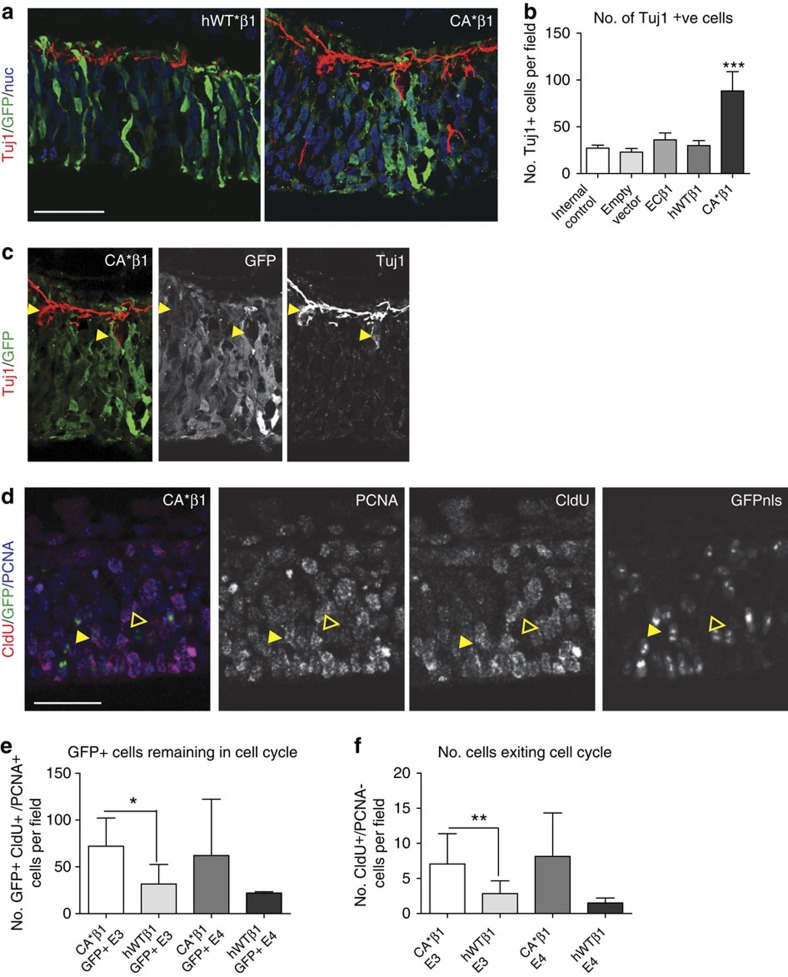
CA*β1 increases neurogenesis in a non-cell autonomous manner. (**a**) Immunostaining for GFP and Tuj1 in E4 midbrain neuroepithelium electroporated with hWTβ1 or CA*β1. Note the increase in Tuj1^+^ (red) neurons with CA*β1 expression. Scale bar, 20 μm. (**b**) Quantification of the number of Tuj1^+^ cells per field. *n*>19 (**c**) Immunostaining of Tuj1 and GFP in the CA*β1 image in **a** showing channels split and merged. Scale bar, 20 μm. Filled yellow arrowheads label Tuj1^+^ GFP^−^ cell bodies. (**d**) Immunostaining of GFP, CldU and PCNA staining in midbrain neuroepithelium electroporated with CA*β1 and GFPnls, treated with CldU 12 h before fixation. Filled yellow arrowheads label GFP^+^CldU^+^PCNA^+^ cells. Outlined yellow arrowheads label GFP^−^CldU^+^PCNA^−^ cells. (**e**) Quantification of GFP^+^CldU^+^PCNA^+^ cells at E3. *n*>5. (**f**) Quantification of CldU^+^PCNA^−^ cells at E3. *n*>5. All graphs: mean and s.d., *P<0.05, **P<0.01, ***P<0.001, one-way analysis of variance.

We next examined if the expanded neuroepithelium containing increased dividing progenitors produced an increased number of neurons. Expression of CA*β1 increased the number of Tuj1^+^ cells per field at E4 within the area of electroporation (Fig. 4a,b), more than doubling the numbers present in the other conditions. This increase was maintained 4 days after electroporation at E6 (Supplementary Fig 6). Unexpectedly, this increase in neurogenesis observed at E4 occurred solely in the GFP^−^ cells—the non-electroporated cells neighbouring the CA*β1-expressing GFP^+^ cells (Fig. 4c). By E6, a small number of GFP^+^ Tuj1^+^-double-positive cells were observed in some experiments (Supplementary Fig. 6), confirming GFP^+^ CA*β1-expressing cells were able to generate neurons and excluding toxicity from CA*β1 or GFP expression as a reason for the lack of GFP^+^ CA*β1^+^ Tuj1^+^ cells observed at E4.

The GFP^+^ and GFP^−^ cell populations also showed differences in cell cycle exit and re-entry. Expression of CA*β1 resulted in an increased number of GFP^+^ cells remaining in the cell cycle 24 h later at E3, before any significant morphological changes in the neuroepithelium (Fig. 4d,e and Supplementary Fig. 2), while at the same time there was also an increase in the overall (that is, GFP^+^ and GFP^−^) number of cells exiting the cell cycle (Fig. 4d,f). It follows that a larger number of GFP^−^ cells must be exiting the cell cycle. The differences in GFP^+^ and GFP^−^ cell behaviour that must be responsible indicate a dual role of integrin signalling, promoting the proliferation and differentiation of progenitors in a cell autonomous and non-cell autonomous manner, respectively.

### Transcriptome analysis suggests a role for Wnt signalling

**Figure 5:**
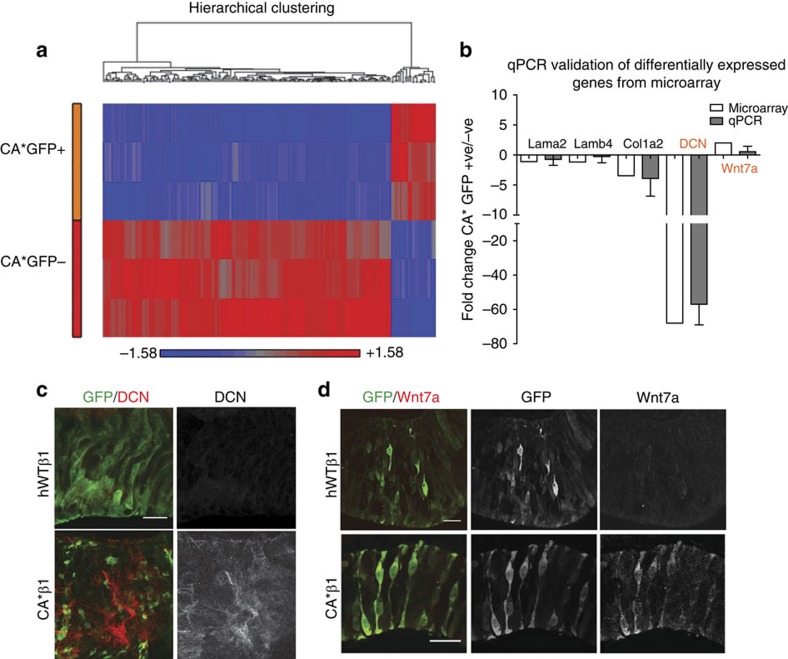
Transcriptome analysis of CA*β1-positive and -negative cells reveals a role for Wnt signalling. (**a**) Heat map showing fold change of gene expression between the CA*β1/GFP^+^ versus CA*β1/GFP^−^ cells. (**b**) Validation of transcriptome results via quantitative PCR. Genes highlighted in red are the genes of interest. (**c**) Immunostaining for GFP and Decorin (DCN) in E4 midbrain neuroepithelium electroporated with hWTβ1 or CA*β1. Scale bar, 20 μm. (**d**) Immunostaining for GFP and Wnt7a in E4 midbrain neuroepithelium electroporated with hWTβ1 or CA*β1. Scale bar, 20 μm.

Having established that enhanced integrin signalling increases neurogenesis, we next asked what signals were responsible. As the increase in Tuj1^+^ cells was seen solely in the GFP^−^ population, we reasoned that cell–cell interactions between CA*β1^+^GFP^+^ and GFP^−^ cells (that is, CA*β1 expressing and non-expressing) are likely responsible for the restriction of neurogenesis to the GFP^−^ cells. To identify candidates for the cell–cell signals, we conducted a transcriptome analysis, comparing the CA*β1^+^GFP^+^ cells and their neighbouring GFP^−^ cells using the Affymetrix Chick Gene 1.0 ST Array. We analysed the RNA from E4 midbrains, electroporated at E2, from three biological replicates. Each replicate was itself a pool of 5–8 embryos. For each embryo, the electroporated area of the midbrain was dissected carefully to ensure that the GFP^−^ cells were only those neighbouring the GFP^+^ cells and not from distant non-electroporated areas. We observed significant differences in gene expression between CA*β1^+^GFP^+^ and GFP^−^ cells (Fig. 5a), with 140 genes showing a twofold change between these two populations (*P*<0.05, *t*-test; data available via GEO database—accession number GSE56632).

Of the 140 genes only 1 was found to be upregulated above a twofold change in the CA*β1^+^ GFP^+^ cells—Wnt7a (Fig. 5b; Table 1). Wnt7a is known to be expressed in the developing neuroepithelium and has numerous roles in neurogenesis, promoting both proliferation and differentiation^3^,^24^,^25^,^26^. Wnt7a and its receptors frizzled 5 and 8 are expressed within the VZ of both mouse and chick embryos (see ref. 3 and GEISHA *in situ* database). Of the larger number of genes found to be upregulated in the GFP^−^ cells, the most highly upregulated gene was the ECM component Decorin (Fig. 5b and Table 2). Decorin is widely expressed during development throughout the CNS^27^ and has numerous roles in the regulation of ECM formation, cell proliferation and differentiation in both the embryo and adult. It interacts with key signalling pathways active in neurogenesis, such as Wnt and transforming growth factor (TGF)-β (refs 28, 29, 30, 31).

### Expression of Wnt7a and Decorin in midbrain neuroepithelium

The transcriptome results showing increased expression levels of both Wnt7a and Decorin in the presence of CA*β1 were confirmed by both quantitative reverse transcription–PCR (qRT–PCR; Fig. 5b) and immunohistochemistry (Fig. 5c,d). Expression of both Wnt7a and Decorin has been previously reported during neural development of the chick embryo. Wnt7a is expressed at the time of electroporation (E2–4; GEISHA *in situ* database), but Decorin is expressed only at earlier stages of neural fold development and neural tube closure^27^. In keeping with this, relatively low levels of Decorin were observed in embryos expressing the other constructs, such as hWTβ1 (Fig. 5c), whereas relatively higher levels of Wnt7a were observed (Fig. 5d).

### Wnt7a and Decorin promote neurogenesis

**Figure 6:**
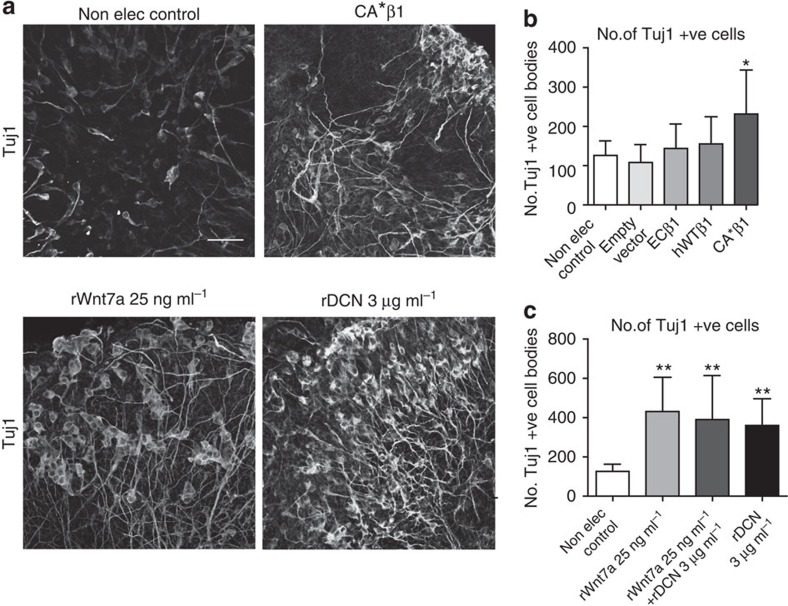
Wnt7a and Decorin promote neurogenesis. (**a**) Immunostaining for Tuj1 in explants—a non-electroporated control, one expressing CA*β1 and non-electroporated explants with recombinant Wnt7a or Decorin (DCN) added. Scale bar, 50 μm. (**b**) Quantification of the number of Tuj1^+^ cell bodies in explants electroporated with the different itgβ1 constructs. *n*>7. (**c**) Quantification of the number of Tuj1^+^ cell bodies in non-electroporated explants with recombinant Wnt7a or DCN added. *n*>5. All graphs: mean and s.d., *P<0.05, **P<0.01, one-way analysis of variance.

To confirm the roles of Wnt7a and Decorin in the integrin-mediated increase in neurogenesis, we used a chick midbrain explant culture system. This allowed pharmacological manipulation of neurogenesis, via the addition of recombinant proteins to the culture media, while maintaining the 3D microenvironment of the tissue. First, the increase in Tuj1^+^ cells from the expression of CA*β1 was confirmed in the explant cultures (Fig. 6a,b). In explants expressing hWTβ1 and other control constructs, levels of neurogenesis were no greater than the non-electroporated control explants. When recombinant Wnt7a or Decorin was added to explants there was a significant increase in the number of Tuj1^+^ cells (Fig. 6a,c), indicating both promote neurogenesis. Interestingly, there was no further increase in Tuj1^+^ cells when both proteins were added in combination (Fig. 6c), suggesting that Wnt7a and Decorin promote neurogenesis via a common pathway.

### Wnt7a/Decorin knockdown blocks CA*β1-driven neurogenesis

**Figure 7:**
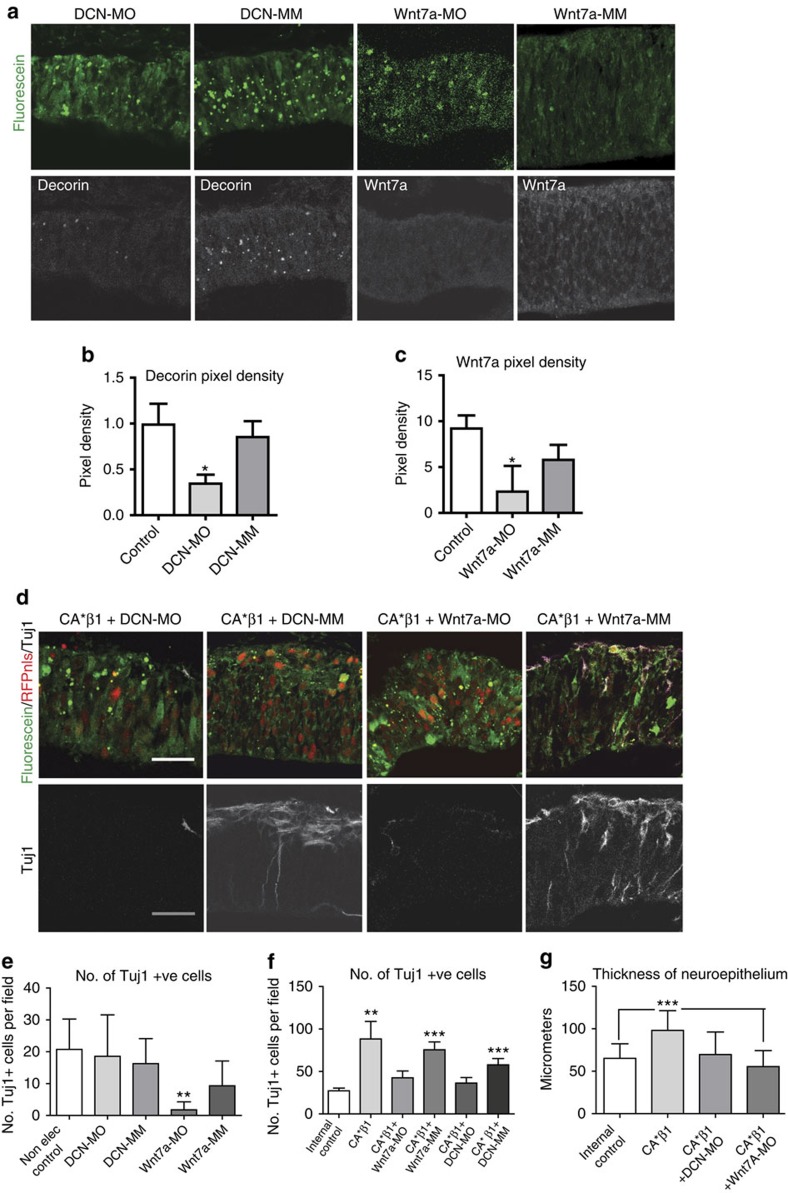
Genetic knockdown of Wnt7a and Decorin blocks CA*β1-mediated neurogenesis. (**a**) Immunostaining for fluorescein to show uptake of the morpholinos (MO) in a control E4 midbrain neuroepithelium electroporated with the DCN-MO, DCN-MM, Wnt7a-MO or Wnt7a-MM morpholinos. (**b**,**c**) Quantification of Decorin and Wnt7a staining intensity in E4 midbrain neuroepithelium after electroporation of morpholinos. *n*>3. (**d**) Illustration by immunohistochemistry for fluorescein, RFPnls and Tuj1 in E4 midbrain neuroepithelium co-electroporated with CA*β1 and a morpholino (either DCN-MO, DCN-MM, Wnt7a-MO or Wnt7a-MM). (**e**) Quantification of the number of Tuj1^+^ cells in E4 midbrain neuroepithelium electroporated only with the morpholinos. Note the loss of neurogenesis with the Wnt7a-MO. *n*>5. (**f**) Quantification of the number of Tuj1^+^ cells in E4 midbrain neuroepithelium co-electroporated with CA*β1 and a morpholino. Note the loss of the significant increase in neurogenesis resulting from CA*β1 expression with both the Wnt7a-MO and the DCN-MO. *n*>8. (**g**) Quantification of the thickness of the neuroepithelium in E4 midbrain neuroepithelium co-electroporated with CA*β1 and a morpholino. *n*>4. Scale bars, 20 μm. All graphs: mean and s.d., *P<0.05, **P<0.01, ***P<0.001, one-way analysis of variance.

To confirm the role of Wnt7a and Decorin in CA*β1-enhanced neurogenesis *in vivo*, and to explore the role of these signalling molecules in normal development, morpholinos (MO) were used to knockdown the genes *in ovo*. Recent studies have shown MO to be a more effective method of gene knockdown in the chick embryo than RNA interference^32^. Splice blocking MO were used, targeting early exon–intron boundaries in both genes. Efficacy was confirmed by PCR of RNA extracted from FACS-purified cells. Both MO created PCR products of sizes that differed to those in untreated cells and in cells targeted by the 5-base mis-match (MM) control MO, suggesting abnormalities of splicing because of the blocking of normal splice sites (Supplementary Fig. 7). For electroporation, the MO were fluorescein tagged to provide both the charge required for entry into the neuroepithelium and a method for the identification of cells targeted by the MO. This revealed that the majority of the cells within the electroporated regions were targeted by the MO (Fig. 7a and Supplementary Fig 7).

The experimental MO produced a significant loss of Wnt7a and Decorin expression, whereas the MM controls had no significant effect (Fig. 7a–c). Expression of the Wnt7a-MO also reduced Tuj1^+^ cells in the control experiments (Fig. 7e). Both the Wnt7a-MO and DCN-MO blocked the increase in Tuj1^+^ cells, the increase in sub-apical PH3^+^ cells (Fig. 7d,f and Supplementary Fig. 7) and the increased thickness of the neuroepithelium observed with expression of CA*β1 (Fig. 7d,g), whereas the MM controls had no effect. These results show that Wnt7a and Decorin are necessary for the neurogenesis enhanced by CA*β1 expression and, consistent with high levels of expression in wild-type E4 midbrain neuroepithelium, that Wnt7a is also required for normal neurogenesis at E2–4.

### Definition of the itgβ1/Wnt7a/decorin pathway

To establish the sequence of signals by which integrin, Wnt7a and Decorin promote neurogenesis, we exposed the midbrain explants electroporated with CA*β1 to a series of small-molecule inhibitors and antibodies to block signalling by each.

**Figure 8:**
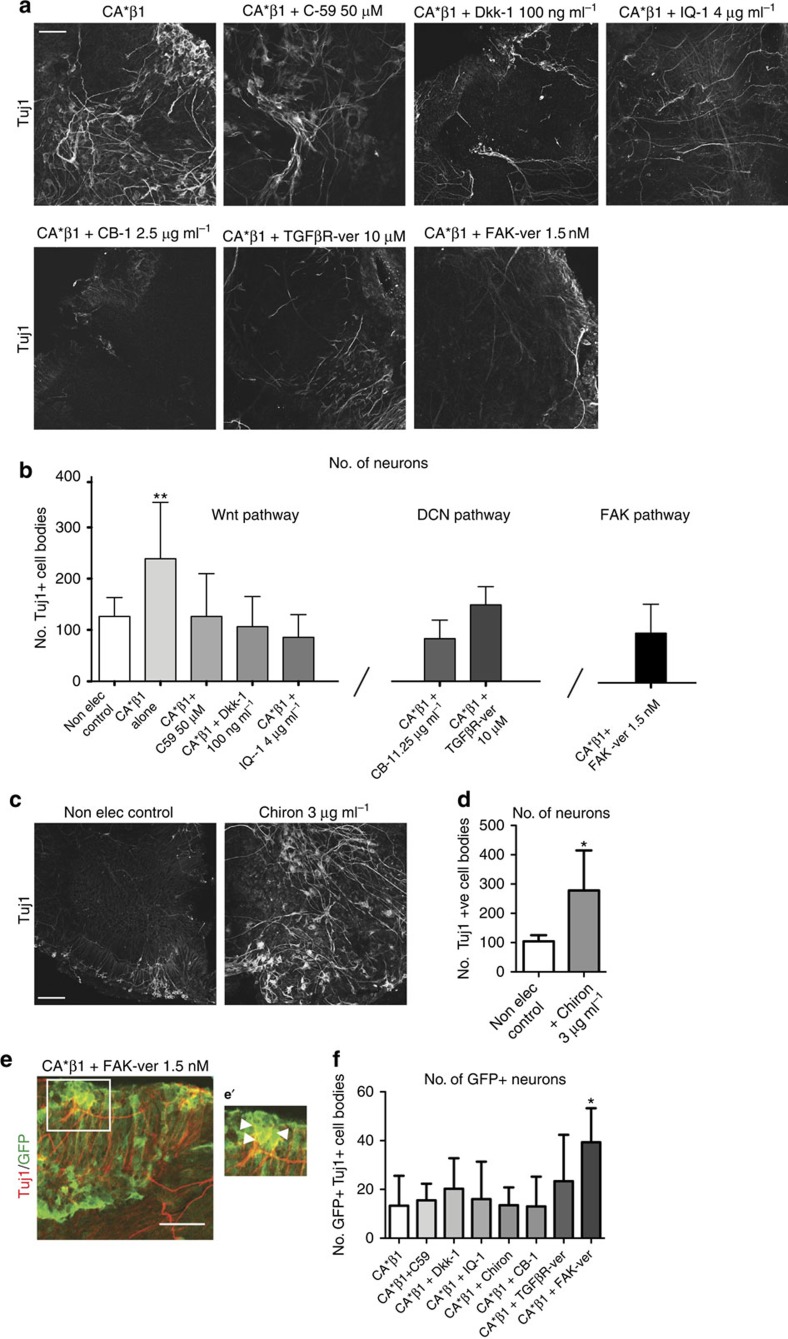
Pharmacological inhibition of integrin, Wnt7a and Decorin activity in explants. (**a**) Immunostaining for Tuj1 in explants expressing CA*β1 alone or treated with Wnt inhibitors C-59, Dkk-1, IQ-1, DCN-blocking antibody CB-1, TGF-βR inhibitor or FAK inhibitor. Scale bar, 50 μm. (**b**) Quantification of the number of Tuj1^+^ cells in explants expressing CA*β1 alone or treated with the inhibitors. Note that all the inhibitors prevent the significant increase in neurogenesis resulting from CA*β1 expression. *n*>4. (**c**) Immunostaining for Tuj1 in non-electroporated explants alone and with the addition of Chiron. (**d**) Quantification of the number of Tuj1^+^ cells in explants treated with Chiron. *n*>4. (**e**) Immunostaining for GFP and Tuj1 in an explant expressing CA*β1 treated with a FAK inhibitor. White box outlines the area of the image in **e**'. Scale bar, 50 μm. (**e**') Enlarged area of **e**, white filled arrowheads label GFP^+^Tuj1^+^ cell bodies. (**f**) Quantification of GFP^+^Tuj1^+^ cells in explants expressing CA*β1 and treated with inhibitors. *n*>5. Mean and s.d. *P<0.05. *n*>4, one-way analysis of variance.

First, we manipulated FAK, a major effector of integrin signalling. Addition of an inhibitor of FAK activity, an ATP-competitive inhibitor PF-562271, blocked CA*β1-enhanced neurogenesis (so returning neurogenesis to wild-type levels as shown in Fig. 8a,b) but had no effect on the pro-neurogenic effects of recombinant Decorin or Wnt7a (Supplementary Fig 8). In the CA*β1+FAK inhibitor explants, expression of Decorin in the GFP^−^ cells showed a fivefold reduction (mean=5.591, s.d.=6.182) and expression of Wnt7a in GFP^+^ cells showed a 15-fold reduction (mean=15.08, s.d.=6.075), as measured by qRT–PCR. Together, these results confirm that both Wnt7a and Decorin act downstream of integrin signalling.

Next, we examined Wnt signalling using C-59, an inhibitor of Wnt secretion via blocking the palmitoylation of Wnts by Porcupine, preventing their secretion^33^. As expected from the MO experiments, addition of C-59 to explants expressing CA*β1 blocked the pro-neurogenic effects of CA*β1 (Fig. 8a,b). Addition of C-59 also reduced the expression of Decorin in the GFP^−^ cells 30-fold (mean=29.90, s.d.=21.21), showing Wnt signalling is upstream of Decorin. Confirming this, the pro-neurogenic effect of recombinant Decorin was not inhibited by C-59 (Supplementary Fig. 8).

Wnt signalling can act via canonical or non-canonical pathways, both of which are blocked by C-59. To distinguish whether our phenotype was beta-catenin dependant (the canonical pathway) or if it was acting through another, non-canonical arm of the pathway, such as nuclear factor of activated T cells (NFAT) or Jun/Fos, we used recombinant Dickkopf-1 (Dkk-1), an antagonist of canonical Wnt signalling via binding to the LRP6 component of the Wnt receptors^34^. Addition of Dkk-1 blocked the CA*β1-mediated neurogenesis (Fig. 8a,b), showing that Wnt7a acts via the canonical signalling pathway. In confirmation of this, Chiron 99021, an inhibitor of GSK-3β which promotes increased stabilization of beta-catenin, resulted in an increase in Tuj1^+^ neurons when added to non-electroporated explants showing stabilization of beta-catenin is sufficient for neurogenesis (Fig. 8c,d).

Beta-catenin regulates Wnt-mediated changes in gene expression by associating with two co-factors; p300 that promotes differentiation^35^,^36^ and Creb-binding protein (CBP) that promotes self-renewal^37^. The small-molecule inhibitor IQ-1 was used to prevent its binding to p300, blocking the increased neurogenesis observed upon expression of CA*β1 (Fig. 8a,b). In contrast, the addition of ICG-001, a small-molecule inhibitor that blocks beta-catenin binding to CBP had no effect on neurogenesis (Supplementary Fig. 8). IQ-1 also reduced the expression of Decorin in the GFP^−^ cells sixfold (mean=6.452, s.d.=3.777), showing that Wnt signalling via the p300 co-activator is required for the upregulation of Decorin and for neuronal differentiation.

To block signalling via Decorin, we used the CB-1 antibody, previously shown to inhibit Decorin function in the chick embryo^27^. Addition of CB-1 blocked the CA*β1-mediated neurogenesis (Fig. 8a,b). Decorin is known to interact with the TGF-β pathway, modulating TGF-β signalling^31^,^37^, and the TGF-β pathway is known to play a role in midbrain development and the regulation of differentiation^38^. To see if TGF-β signalling is required for Decorin-mediated neurogenesis, an inhibitor of both TGF-βR1 and 2, LY2109761 (TGF-βR-ver), was added to explants. Addition of LY2109761 to non-electroporated explants alongside recombinant Decorin blocked the increase in neurogenesis (Supplementary Fig. 8). LY2109761 also blocked the increase in neurogenesis observed after CA*β1 expression (Fig. 8a,b). These data show that Decorin is regulating neurogenesis via the TGF-β signalling pathway.

### Inhibition of FAK activity increases CA*β1 GFP^+^ neurons

Finally, we asked why Tuj1^+^ cells were generated only by the GFP^−^ cells, when both Wnt7a and Decorin are secreted factors able to signal to both GFP^+^ and GFP^−^ cells. We noted that addition of the FAK inhibitor PF-562271 had the effect, in addition to reducing neurogenesis in the presence of CA*β1 as described above, of increasing the generation of GFP^+^ Tuj1^+^ cells in the same explants (Fig. 8e,f). FAK activity downstream of integrin signalling may therefore both promote Wnt7a secretion and block differentiation. In contrast, blocking Decorin function or the Wnt pathway via any of the other inhibitors did not result in the generation of GFP^+^ Tuj1^+^ cells (Fig. 8f), although the CBP inhibitor ICG-001 reduced the number of GFP^+^ CA*β1^+^ cells within the explants (Supplementary Fig. 8), suggesting Wnt signalling in the CA*β1 cells may also prevent differentiation via the CBP co-activator promoting the self-renewal of these cells.

## Discussion

Using the chick as a model system, we have examined the cellular and molecular consequences of integrin signalling in NSCs. We have used electroporation of an activated integrin as a strategy to overcome drawbacks of previous studies using knockouts, blocking or activating antibodies. We report two consequences of enhancing integrin signalling within the neuroepithelial cells of the midbrain or mesencephalon. First, a cell autonomous effect of increased proliferation and inhibited differentiation of cells expressing the activated integrin, leading to the appearance of cells with the properties of SAP cells as defined in the mouse. Second, a non-cell autonomous effect of greater levels of neurogenesis in the adjacent, non-integrin-expressing, cells driven by Wnt7a and the consequent upregulation of Decorin.

The significance of our findings can be split into two parts—that which defines the downstream signalling pathways as to how integrins enhance neurogenesis in the chick neuroepithelium and that which demonstrates the consequences of an increased rate of proliferation for the architecture of the neuroepithelium. Regarding the former, our finding that the Wnt7a morpholino reduced neurogenesis in wild-type embryos is in keeping with a role the integrin-Wnt7a pathway during the normal development of the chick CNS. This role of Wnt7a in integrin-mediated neurogenesis is further supported by the previously reported effects of the protein; Wnt7a can regulate the asymmetry of spindles in neuroepithelial cells of the VZ, linked to asymmetric cell division^25^, and studies in knockout mice have shown Wnt7a promotes both NSC proliferation and differentiation in the adult hippocampus^26^,^39^ and in the embryonic ventral midbrain where Wnt7a^−/−^ mice show reduced Sox2^+^ progenitors in the VZ^40^.

**Figure 9:**
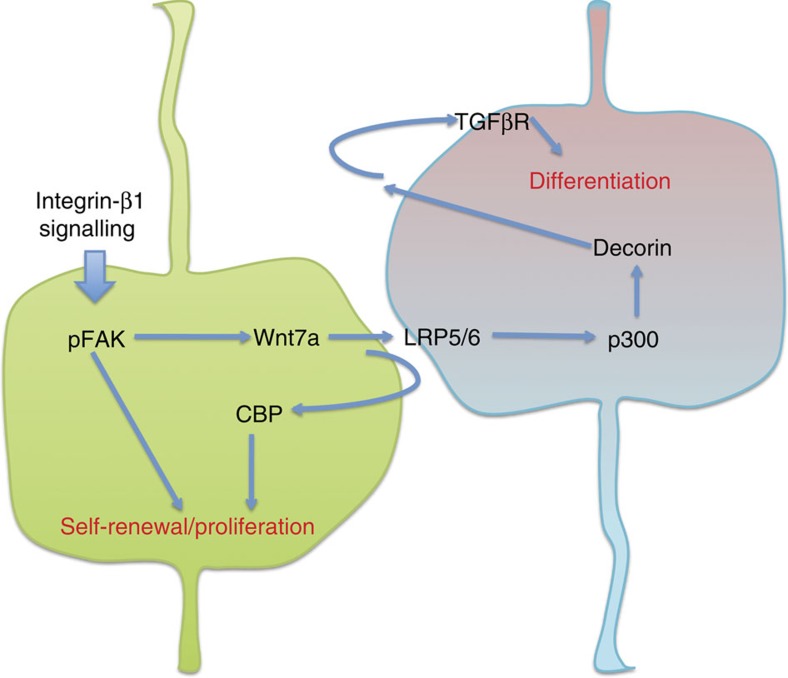
Model of the integrin-β1–Wnt7a–Decorin pathway. Schematic showing the proposed model of integrin-β1–Wnt7a–Decorin signalling pathway. The green cell represents the CA*β1-expressing cell, which undergoes proliferation and self-renewal with upregulation of Wnt7a. Wnt7a is secreted, promoting Wnt7a signalling within the CA*β1-expressing cell via the CBP branch of the Wnt pathway, leading to proliferation and self-renewal. Wnt7a also acts on the neighbouring, negative cell (blue/red cell) via the LRP5/6 receptor, signalling via the p300 branch of the Wnt pathway. This leads to the upregulation of Decorin expression. Decorin is secreted and acts in an autocrine manner on the negative cell via the TGF-β receptor, promoting differentiation.

The differences observed between the CA*β1^+^ and CA*β1^−^ cells in response to the inhibitors ICG-001 and IQ-1 suggests that distinct Wnt signalling pathways may contribute to the behaviours of the two cell populations. As ICG-001 had no effect on CA*β1-mediated neurogenesis, but did reduce the number of GFP^+^ cells, this would suggest Wnt signalling in the CA*β1^+^ cells is driving self-renewal via the CBP co-activator. Conversely, the inhibition of neurogenesis by the p300 inhibitor IQ-1 suggests that, within the neighbouring CA*β1^−^ cells, Wnt7a signals via the p300 co-activator to both promote the upregulation of Decorin and the differentiation of the cells. Decorin reinforces this differentiation via the TFG-β pathway, resulting in the increased neurogenesis observed. This model (Fig. 9) allows for both the cell autonomous and non-autonomous effects of CA*β1 signalling, which promotes the different responses of the CA*β1^+^ and CA*β1^−^ cells to the secreted factors Wnt7a and Decorin. The role of Decorin in this model increasing differentiation contrasts with recent findings in the kidney^28^, where Decorin released by differentiating cells inhibits the Wnt-mediated differentiation of neighbouring cells. However, Decorin has multiple interactions in addition to that with the TGF-β pathway observed in our study, including modulation of Met receptor function as well as acting as a link protein for collagens^31^,^41^,^42^,^43^ and functions in different tissues may therefore be cell-type specific.

Turning to the latter—the consequences of increased proliferation—the appearance of sub-apically dividing cells (a cell population not normally present in the chick neuroepithelium at this stage) within an architecturally normal neuroepithelium likely reflects the increased number of dividing cells, which, without lateral expansion of the neuroepithelium, are competing for space to divide within the same apical ventricular area. Pseudostratification of the neuroepithelium, where the nucleus drops down to the ventricular surface only to undergo mitosis, provides a mechanism to maximize the use of apical space but ceases to be advantageous if cells are having to wait to undergo mitosis, lengthening the cell cycle. One way to overcome this is to allow cells to undergo mitosis sub-apically, a mechanism observed by Pilz *et al*.^44^ in the mouse LGE with the generation of SAPs. They suggest SAPs are the initial step in overcoming apical constraint and an intermediate step in the generation of the many types of proliferative BPs found in the mammalian neocortex^44^,^45^. Both types of progenitors enable expansion of the neuroepithelium without using the ventricular surface for mitosis, thus over-coming apical constraint. However, BPs divide away from the VZ, in the various parts of the SVZ, whereas SAPs divide within the basal portion of the VZ, maintaining apical attachment. We propose, therefore, that the high levels of proliferation generated by integrin signalling in our experiments lead to the formation of SAPs, as normally seen in mammalian brains with greater levels of proliferation than in the chick.

Although Decorin does have a necessary role earlier in the development of the chick nervous system, where blocking function via an antibody resulted in defects of neural tube closure and disorganized layers and tears within the neuroepithelium^27^, it is not normally expressed in the chick neuroepithelium between E2 and E4. In our experiments, expression is induced by integrin signalling and is linked to the appearance of the SAPs, as Decorin MO prevented the appearance of SAPs in the explants expressing CA*β1. The conclusion that Decorin is therefore linked to the emergence of cell populations characteristic of the more complex mammalian CNS is supported by the work of Pilz *et al*.^44^, who observed that SAPs are increased in the VZ and cells with a similar morphology to SAPs—bipolar radial glia cells dividing sub-apically—were also increased in the outer subventricular zone (OSVZ) in the ferret—an area which, in humans, is enriched for ECM and integrins^15^, and is the only area of the human neocortex to express Decorin (data set from Fietz *et al*.^15^, GEO accession GSE38805). Within the OSVZ, progenitors such as SAPs undergo self-renewing divisions as well as differentiation, unlike the neighbouring inner subventricular zone that contains BPs that mainly differentiate without self-renewing. The high levels of Decorin expression within the OSVZ compared with the inner subventricular zone could instruct these important differences in neurogenic potential and so play a role in the expansion of progenitors and thickening of the OSVZ observed in gyrencephalic cortices.

We conclude, therefore, that our study identifies a potential role for integrins and Decorin in the complex mammalian CNS that is not normally utilized in the chick. Certainly, the areas of highest self-renewal in the developing mammalian cortex, the VZ and OSVZ, contain the highest levels of ECM and integrin expression, suggesting higher levels of integrin signalling^15^. In experiments to explore this hypothesis, simply documenting integrin levels will not be sufficient. Integrins are highly stable at the cell surface^46^,^47^,^48^,^49^ and it is unlikely that surface levels will change with a speed sufficient to allow fluctuations in signalling. However, the activation state of integrins can be altered very quickly^50^,^51^,^52^,^53^,^54^, allowing changes in signalling to occur rapidly, and studying the activation states of integrins within the neuroepithelium would be important in understanding the effects of signalling within the different proliferating and differentiating zones of the mouse and human cortex.

## Methods

### *In ovo* electroporation and explant culture

Fertilized chicken (Gallus gallus domesticus) eggs were obtained from the Poultry Research Institute (Roslin, the University of Edinburgh). Eggs were incubated at 38 °C for 45 h to the desired Hamburger-Hamilton stage^55^. Midbrains were electroporated with 1.0–2.0 μg ml^−1^ plasmid DNA (see below for plasmids). Explants were dissected immediately after electroporation; midbrains were dissected and manually cut along the ventral side to flatten for culture. Explants were placed ventricle side down on floating membranes (Whatman Nucleopore membranes). Culture conditions were adapted from Das *et al*.^56^; neurobasal media were supplemented with 1% penicillin and streptomycin, 1% Glutamax and 2% B-27, explants were cultured at 37 °C at 5% CO_2_.

Recombinant human Wnt7a protein (Abcam, ab116171), used at 50 ng ml^−1^ and 25 ng ml^−1^ concentration, and recombinant human Decorin protein (R&D, 143-DE), used at 1 μg ml^−1^ and 3 μg ml^−1^ concentration, were added to the media of explant cultures at the appropriate concentrations for the total length of culture. An ATP-competitive, reversible inhibitor of FAK (Selleckchem PF-562271), used at the manufacturer's recommended IC_50_ concentration of 1.5 nM, and C-59 an inhibitor of Wnt secretion, via prevention of palmitylation of Wnt by Porcupine (Cellagen Technology, C7641), used at the manufacturer's recommended IC_50_ concentration of 50 μM, were also added to culture media as above. The CB-1 anti-Decorin antibody (DSHB) was added to the culture media at a concentration of 1.25 μg ml^−1^ and 2.5 μg ml^−1^ as previously described^27^. The small-molecule inhibitors of Wnt signalling IQ-1 (Tocirs, 4713) and ICG-001 (Tocris, 4505) were used at their recommended concentrations of 4 μg ml^−1^ and 10 μg ml^−1^, respectively. Chiron 99021 (Tocris, 4423), an inhibitor of GSK-3β, was used at the recommended concentration of 3 μg ml^−1^.

### Plasmids

Plasmids were constructed as described in Laursen *et al*.^23^. The CA* itgβ1 contained the previously published D723R mutation^22^,^23^ and the EC only itgβ1 is a truncated version of the human WT itgβ1, containing only the extracellular and transmembrane domains. The vector used for all constructs and as the empty vector control is pcDNA 3.1 (−). Cytoplasmic and nuclear-localized EGFP (GFPnls) plasmids and a nuclear RFP plasmid (kind gift of Federico Calegari, Centre for Regenerative Therapies, TU Dresden, Germany) were also used.

### Immunohistochemistry and analysis

Tissues were fixed in 4% PFA at 4 °C for 1 h before embedding for cryosectioning or prepared for free-floating staining. Primary antibodies used: GFP (1/500, Abcam, ab13970), CldU 5-Chloro-2′-deoxyuridine (1/200, Serotec, OBT0030CX), Decorin (1/100, DSHB, CB-1), FAK (1/200, BD-Biosciences, 610087), pFAK^Y397^ (1/200, Invitrogen, 44625G), fluorescein (1/500, Abcam, ab6655), huβ1 (1/100, Millipore, MAB1981), N-cadherin (1/200, Sigma, C3678), proliferating cell nuclear antigen (PCNA) (1/200, Dako, M0879), PH3 (1/200, Millipore 06-570), Streptavidin-488 (1/1,000, Life technologies, S-11223), Tuj1 (1/500, Covance, MMs-435 P), Tuj1 (1/500, Abcam, ab18207), Wnt7a (1/100, Abcam, ab100792). All secondary antibodies were Alexa Flour conjugates (Invitrogen; 1/1,000). Phalloidin Alexa 568 was added with the secondary antibody (Invitrogen, A12380). Images were acquired using × 20, × 40 and × 63 objective on a Leica SPE microscope system. Images were analysed and quantified blind using ImageJ and ImagePro. The centre of the electroporated area of neuroepithelium was selected for imaging in both cryosections and whole mounts. In cryosections, 300 μm of neuroepithelium in this image was used for counting and analysis and in whole mounts the entire field was analysed. Tuj1^+^ neuron counts were performed using the Tuj1 and nuclei channels to allow Tuj1^+^ cell bodies to be counted. For FACS analysis, DF-1 cells were stained live with no permeabilization.

All experiments contain at least three biological replicates. All data sets were analysed for Gaussian distribution before the appropriate parametric or non-parametric statistical test, either *t*-test or analysis of variance. All data were analysed and quantified blinded.

### Cell culture

Chick fibroblast cell line, DF-1 (kind gift of Dr Mike McGrew, Roslin Institute, University of Edinburgh, UK) were cultured in DMEM supplemented with 10% fetal bovine serum, 1% penicillin and streptomycin and 1% non-essential amino acids and cultured at 39 °C and 5% CO_2_. Plasmids were transfected into cells using lipofectamine 2000 transfection reagent. Cells were cultured for 2 days after transfection.

### Microarray, quantitative PCR and sample preparation

Samples for microarray analysis and qRT–PCR were prepared in the same way. Midbrains from embryos electroporated at E2 were dissected at E4; the GFP-positive area was collected from five to eight embryos and dissociated in Acutase to allow FACS separation of the neighbouring GFP-positive and -negative populations. FACS was performed using a BD Biosciences Aria II cell sorter (BD Biosciences). RNA was extracted using a Qiagen RNeasy Micro Kit (Qiagen) and either sent for microarray analysis (Affymetrix Chick Gene 1.0 array, ARK Genomics) or used for cDNA synthesis using the Invitrogen SuperScript First Strand Synthesis system and qRT–PCR was performed using commercially available Qiagen Quantitect primers. The data discussed in this publication have been deposited in the NCBI's Gene Expression Omnibus and are accessible through GEO Series accession number GSE56632 (http://www.ncbi.nlm.nih.gov/geo/guery/acc.cgi?acc=GSE56632 ).

### GEISHA *in situ* database

Data for this paper were retrieved from the GEISHA database, University of Arizona, Tucson, AZ 85724; World Wide Web URL: http://geisha.arizona.edu (refs 57, 58).

### Cell cycle studies

For quitting fraction studies, 100 μl of 5 mg ml^−1^ concentration of CldU was added 12 h before fixation. Cryosections were co-stained for PCNA and the percentage of CldU^+^PCNA^−^ cells was calculated to demonstrate the fraction of cells exiting the cell cycle. For FACS analysis, embryos were prepared as per microarray preparation. Immediately, before analysis, Sony i-Cyt DAPI solution (Sony AE700570) was added to the cells. 10,000 events were recorded per sample and analysed on FlowJo. GFPnls (GFP with a nuclear localisation signal) was used for these experiments to allow visualization of CldU and PCNA.

Quitting fraction was calculated using CldU, given 12 h before fixation, and PCNA, similar to Magnani *et al*.^59^. Cells that remained in the cell cycle (Np) were CldU^+^PCNA^+^ and cells that had left the cell cycle (Nq) were CldU^+^PCNA^−^. The total number of CldU^+^ cells is (Np+Nq). The percentage of cells exiting the cell cycle is calculated as Nq/(Np+Nq).

### Morpholinos

Splice blocking MO against Wnt7a and Decorin were designed and produced by Gene Tools. The Wnt7a morpholino (MO) targets the exon2–intron3 boundary and the Decorin MO targets the intron1–exon2 boundary. Five base MM controls were used for both Wnt7a and Decorin. MOs were fluorescein tagged, allowing electroporation. MOs were used at a concentration of 500 μM for *in ovo* electroporation as described above and similar to Mende *et al*.^32^. For co-electroporation with CA*β1 and RFP, MO final concentration was 500 μM and the same settings were used as above. MOs against Decorin targeted the intron1–exon2 boundary:

MO—5′- AGAACCTACGAACCACAGTAGAGAA -3′,

MM—5′- AGAAACTAAGAAACACAATAGAAAA -3′.

MOs against Wnt7a targeted the exon2–intron3 boundary:

MO—5′- CTAGTAGAGAGGCAACGTACCCACT -3′,

MM—5′- CTACTACAGACGCAACCTAGCCACT -3′.

### Live time-lapse imaging

For the live time-lapse imaging, embryos were electroporated as described earlier. After 24–48 h, *in ovo* following electroporation, the midbrains were dissected as described for explant cultures and then manually sectioned through the area of electroporation, visualized on a fluorescent stereomicroscope. The samples were then treated as previously described in Das *et al*.^56^, but imaging was performed using an Andor Revolution XDi inverted system (based on an Olympus BX83 and a Yokogawa CSU-X1 spinning disk module) and Olympus Universal Plan Super Apochromat objective (air) with × 20 magnification, numerical aperture=0.75. Briefly, after dissection the tissue was embedded in a collagen mix, allowed to set at 37 °C for 1 h before media were added. The tissue was then allowed to recover for a further 2 h before imaging. Imaging was conducted at 37 °C for a minimum of 16 h. Stacks of 521 × 512 pixels were taken at 140 intervals of 0.5 microns every 5 min.

## Additional information

**Accession codes:** The microarray data have been deposited in the NCBI's Gene Expression Omnibus database under accession code GSE56632.

**How to cite this article**: Long, K. *et al*. Integrin signalling regulates the expansion of neuroepithelial progenitors and neurogenesis via Wnt7a and Decorin. *Nat. Commun.* 7:10354 doi: 10.1038/ncomms10354 (2016).

## Supplementary Material

Supplementary Information Supplementary Figures 1-8 and Supplementary Table 1

Supplementary Movie 1 Normal Inter-kinetic Nuclear Migration (INM) in hWTβ1 expressing neuroepithelium. Two cells are identified via a yellow and orange arrow. These cells migrate to the apical surface (bottom of frame) to divide. After division their daughter cells (yellow and orange arrows) migrate basally. The time between each frame is 5 minutes.

Supplementary Movie 2 Normal INM in CA*β1 expressing neuroepithelium. One cell is identified via a yellow arrow. This cell migrates to the apical surface (bottom of frame) to divide. After division the daughter cells (yellow arrows) migrate basally. Many other cells can be seen migrating apically and dividing too. The time between each frame is 5 minutes.

Supplementary Movie 3 CA*β1 expressing cell divides apically and daughter cells re-extend a basal process. One cell is identified via a yellow arrow. This cell migrates to the apical surface (bottom of frame) to divide. After division the daughter cells (yellow arrows) migrate basally and re-extend a basal process each (smaller yellow arrowheads), reaching the basal surface. The apical processes of the daughter cells are also observed, attached to the apical surface. The time between each frame is 5 minutes.

Supplementary Movie 4 CA*β1 expressing cell divides basally and daughter cells re-extend an apical process. One cell is identified via a yellow arrow. This cell divides in a sub-apical position. After division the daughter cells (yellow arrows) re-extend an apical process each (smaller yellow arrowheads). The basal processes of the daughter cells are also observed, attached to the basal surface. The time between each frame is 5 minutes, the first two frames are paused to allow better visualisation of the division.

Supplementary Movie 5 CA*β1 expressing cells divide apically and sub-apically simultaneously. Cells migrate to divide apically (cyan arrow) or divide basally (yellow arrow) simultaneously within the CA*β1 expressing neuroepithelium. Their daughter cells are identified with the respective colour arrows. The time between each frame is 5 minutes.

## Figures and Tables

**Table 1:** Genes upregulated in GFP^+^CA*β1^+^ cells.

**Gene symbol**	**Gene name**	**Fold change**
DCN	Decorin	67.9917
HBAA	Haemoglobin, alpha 1	57.7155
HBZ	Haemoglobin, zeta	53.4344
LUM	Lumican	47.5684
HBZ	Haemoglobin, zeta	42.812

**Table 2:** Genes upregulated in GFP^−^CA*β1^−^ cells.

**Gene symbol**	**Gene name**	**Fold change**
Wnt7a	Wingless-type MMTV integration site family, member 7A	2.01403
MIR3534	MicroRNA mir-3534	1.83338
MUC13	Mucin 13, cell surface associated	1.76494
EPB41L4A	Erythrocyte membrane protein band 4.1 like 4A	1.70387
PLEKHA8	Pleckstrin homology domain containing, family A	1.70084
