# Supplementary material for: Integrin signalling regulates the expansion of neuroepithelial progenitors and neurogenesis via Wnt7a and Decorin
Source: Nat Commun. 2016 Feb 3;7:10354. doi: 10.1038/ncomms10354 (PMC4742793; doi:10.1038/ncomms10354)
Supplement: Supplementary Information — Supplementary Figures 1-8 and Supplementary Table 1 [file ncomms10354-s1.pdf]

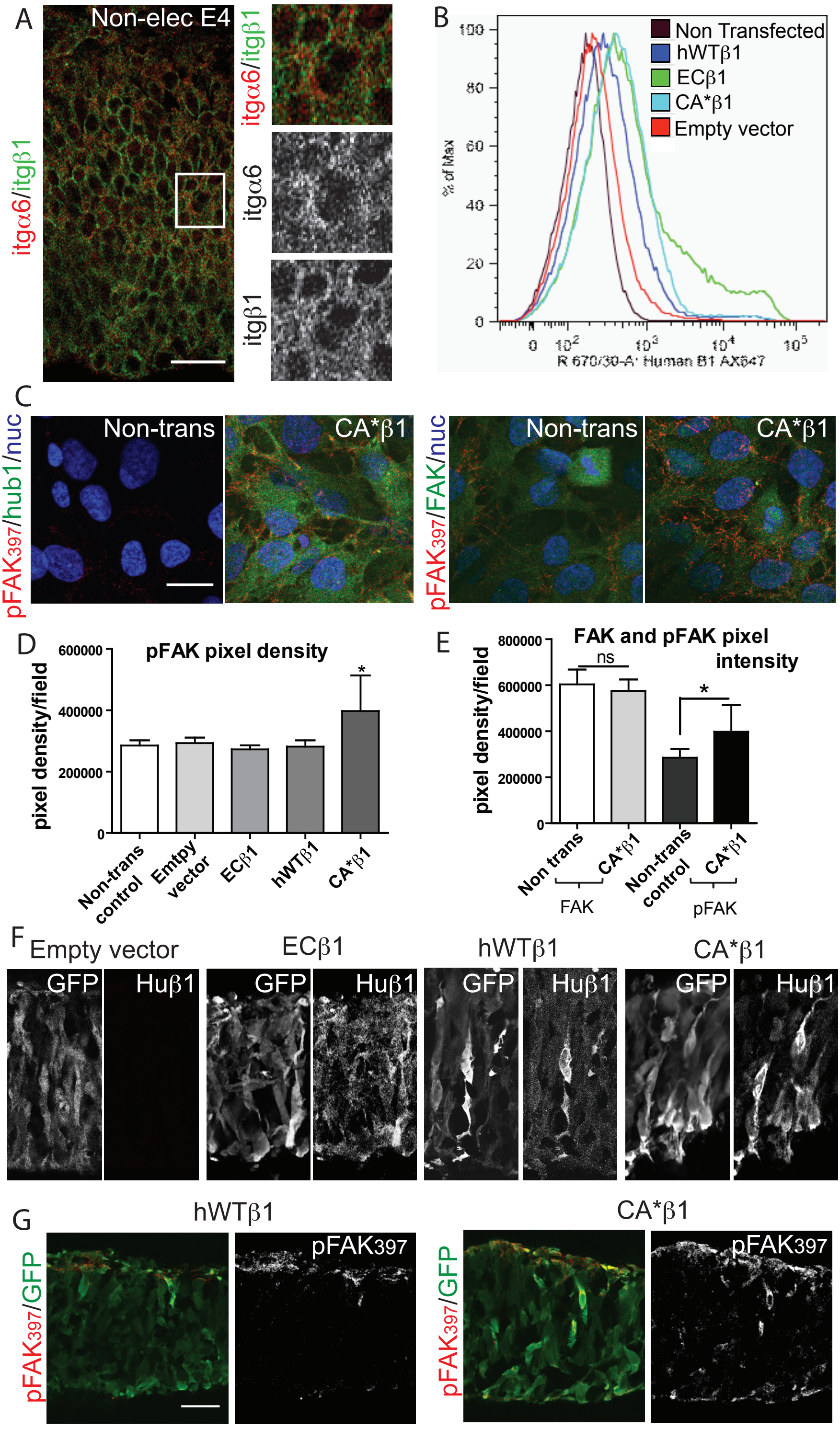

Supplementary Figure 1. Expression and manipulation of itgβ1 signalling within the chick neuroepithelium.
(A) Immunostaining of integrin alpha 6 (itgα6) and β1 (itgβ1) in chick E4 midbrain neuroepithelium. (B) FACS histogram of chick fibroblasts (DF-1 line) stained for human itgβ1(huβ1). DF-1 cells were non transfected or transfected with the empty vector, wild type itgβ1 (hWTβ1), itgβ1 lacking the intracellular domain (ECβ1) and constitutively active (CA*β1) (see legend). (C) Immunostaining for pFAKY397 and huβ1 in non-transfected and CA*β1 expressing DF-1 cells and immunostaining for total FAK and pFAKY397 in non-transfected and CA*β1 expressing DF-1 cells. Scale bar 50μm. (D) Quantification of pFAKY397 pixel density in DF1 cells and (E) compared to total FAK levels. Mean and SD. * = $p<0.05$, One way ANOVA. (F) Immunostaining of GFP and huβ1 in E4 chick midbrain neuroepithelium electroporated with the empty vector, hWTβ1, ECβ1 or CA*β1, all co-electroporated with a cGFP. Scale bar 20μm. (G) Immunostaining of GFP and pFAKY397 in E4 midbrain electroporated with hWTβ1 or CA*β1. Scale bar 20μm.

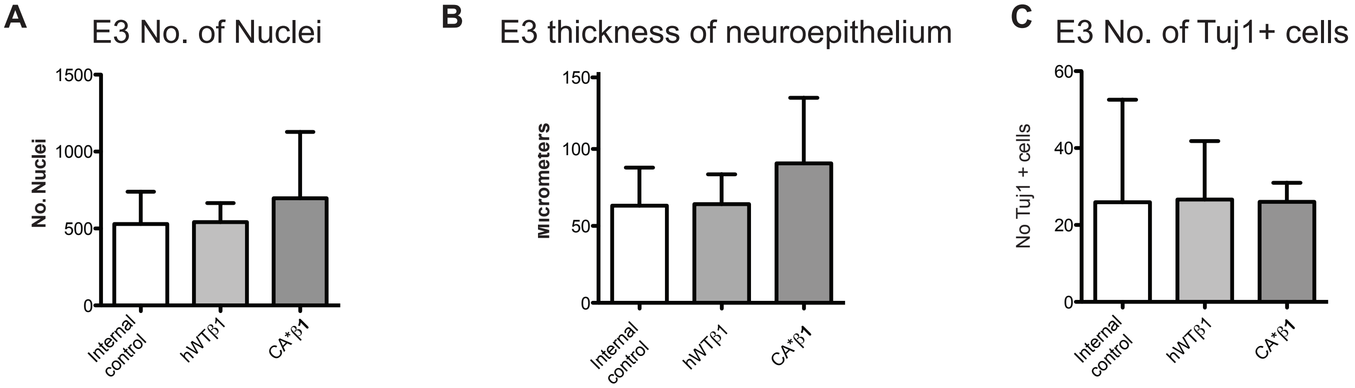

Supplementary Figure 2: Quantification of neuroepithelium at E3. A) Graph showing qunatification of number of nuclei and B) thickness of neuroepithlium and C) number of Tuj1+ neurons. All ns. n>5, mean (SD).

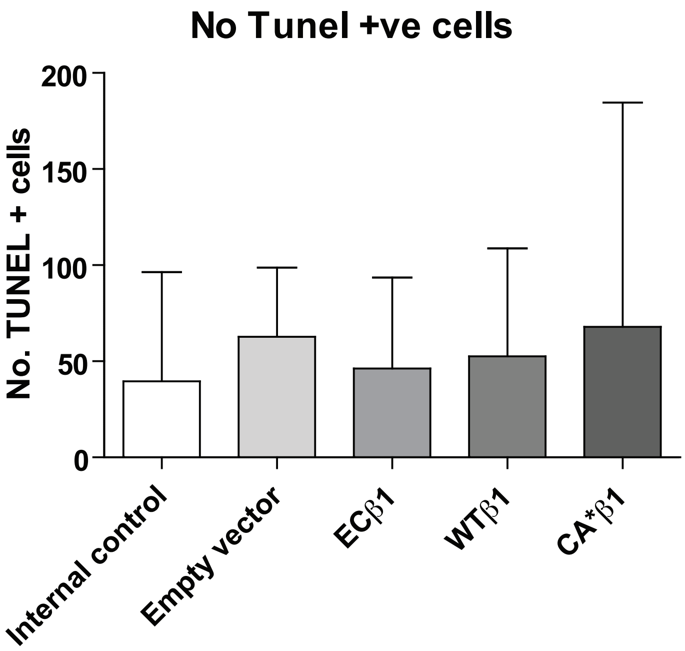

Supplementary Figure 3: TUNEL quantification. Graph showing quantification of TUNEL + cells at E4. All ns. n>14, mean (SD).

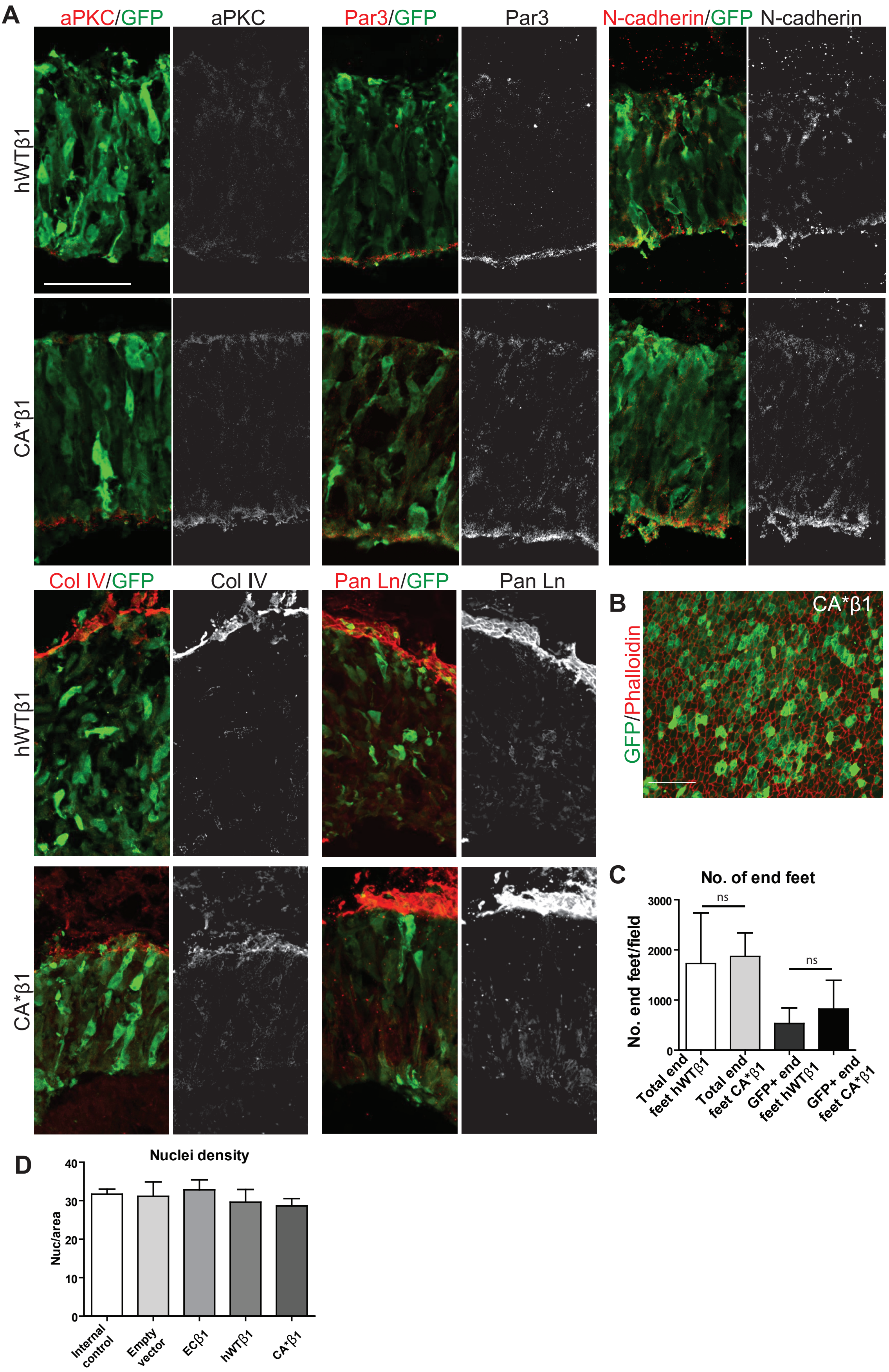

Supplementary Figure 4: Expression of CA*β1 does not effect the polarity of the neuroepithelium.

(A) Immunostaining for GFP and the apical markers aPKC, Par3 and N-cadherin or the basement membrane markers Col IV and Laminin (Pan Ln) in E4 midbrain neuroepithelium electroporated with hWTβ1 or CA*β1. Scale bar 20μm. (B) Immunostaining for GFP and Phalloidin in E4 midbrain neuroepithelium electroporated with CA*β1. Scale bar 50μm. Image shows enface view of ventricular surface of neuroepithelium. (C) Quantification of the number of end feet. Mean and SD. ns = not significant. (D) Quantification of nuclear density in the apical area (5-6 nuclei above the ventricular surface). All ns, n>5, mean (SD).

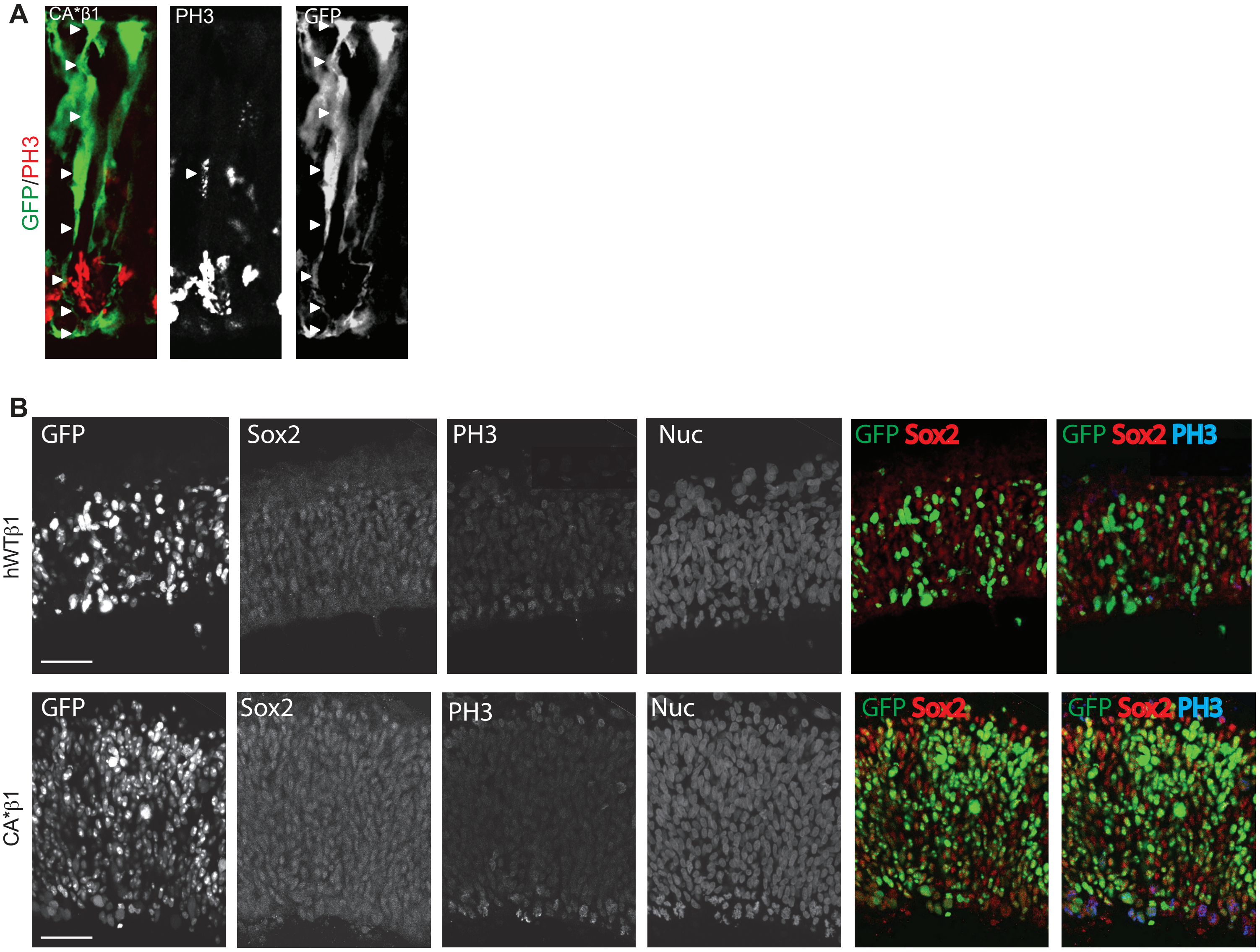

Supplementary Figure 5: Sub-apically dividing CA*β1 expressing cells maintain apical and basal contact and the majority of the neuroepithellium in Sox2+. (A) Immunostaining for GFP and PH3, merge and split channels, showing sub-apical PH3+ GFP+ cells with apical and basal attachment (white arrowheads). (B) Immunostaining for GFP, Sox2, PH3 and Nuc with merges. Scale bar 50μm.

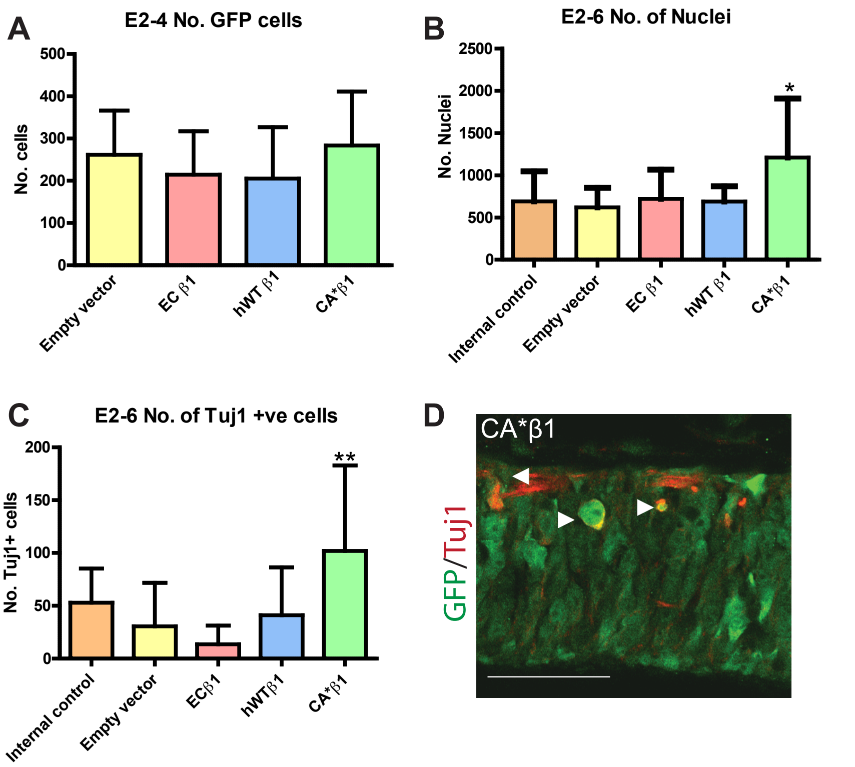

Supplementary Figure 6: Quantification of cells at E4 and E6. A) Number of GFP+ cells at E4. All ns, n>19, mean (SD). B) Number of nuclei at E6 and C) Number of Tuj1+ cells at E6. For both B) and C) n>7, mean (SD). $p<0.05$ (*), $p<0.01$ (**), One way ANOVA. D) GFP and Tuj1 double positive cells at E6 (arrowheads). Scale bar - 50μm.

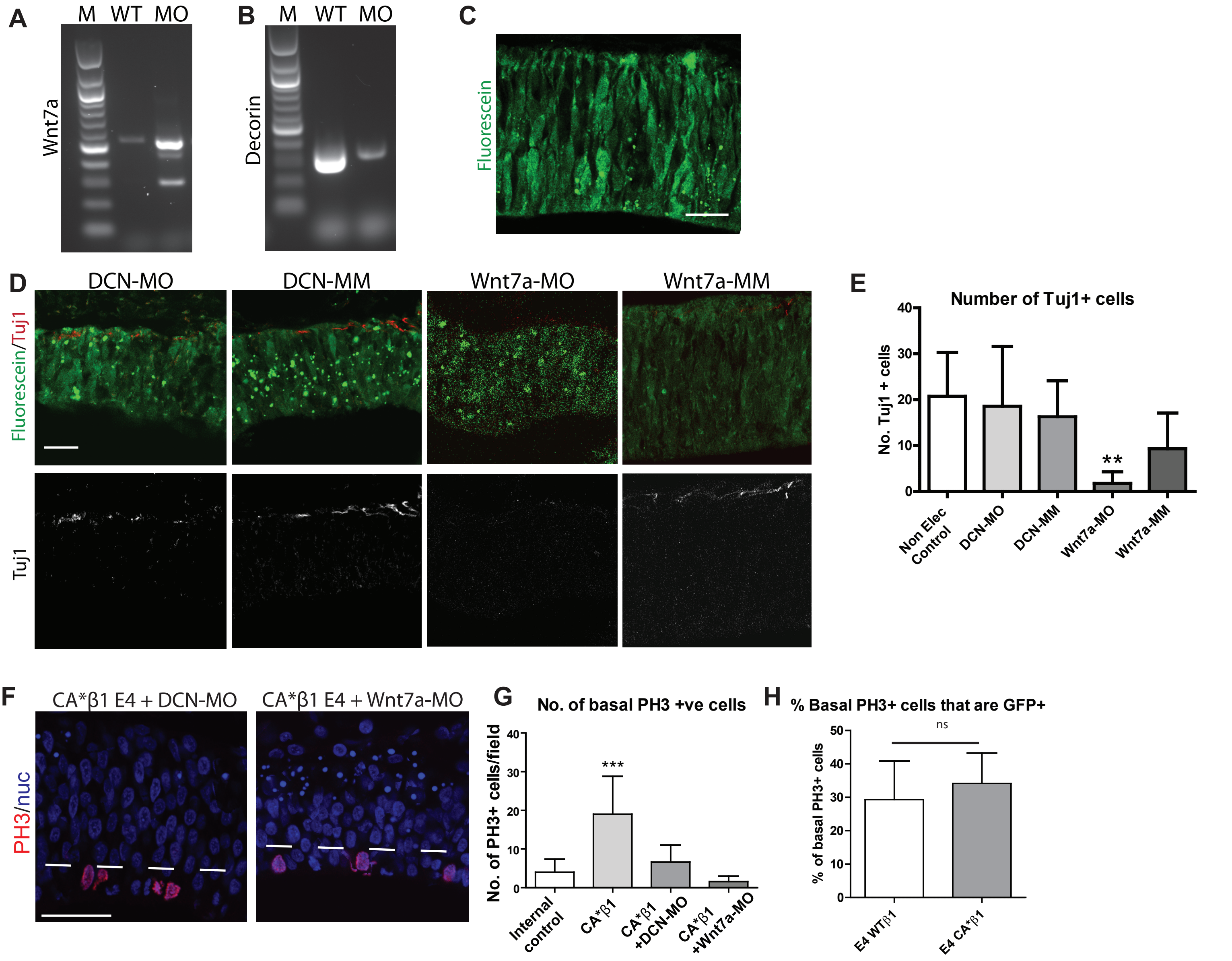

Supplementary Figure 7: Morpholino knockdown of Wnt7a and Decorin in the chick neuroepithelium. (A) RT-PCR products from chick midbrains electroporated with Wnt7a MO demonstrate the absence of WT Wnt7a and the production of several bands with the splice blocking MO targeted to the exon2-intron3 boundary. (B) RT-PCR products from chick midbrains electroporated with DCN MO demonstarte the absence of WT Decorin and the production of a larger band with the splice blocking MO targeted to the intron1-exon2 boundary. (C) Chick midbrain neuroepithelium expressing the fluoroscein tagged MOs. (D) Chick neuroepithelium expressing the MOs immunostained for Tuj1. (E) Quantification of Tuj1+ cells when MOs are expressed. (F) Immunostaining for PH3 in E4 midbrain neuroepithelium co-electroporated with CA*β1 and a morpholino. Dashed white line labels the apical area. (G) Quantification of the number of basal PH3+ cells. (H) Quantification of the percentage of basal PH3+ cells that are GFP+ at E4. Scale bars 20μm. n=5-17 embryos, Mean and SD, ns = not significant, **=p<0.01, ***=p<0.001, One way ANOVA.

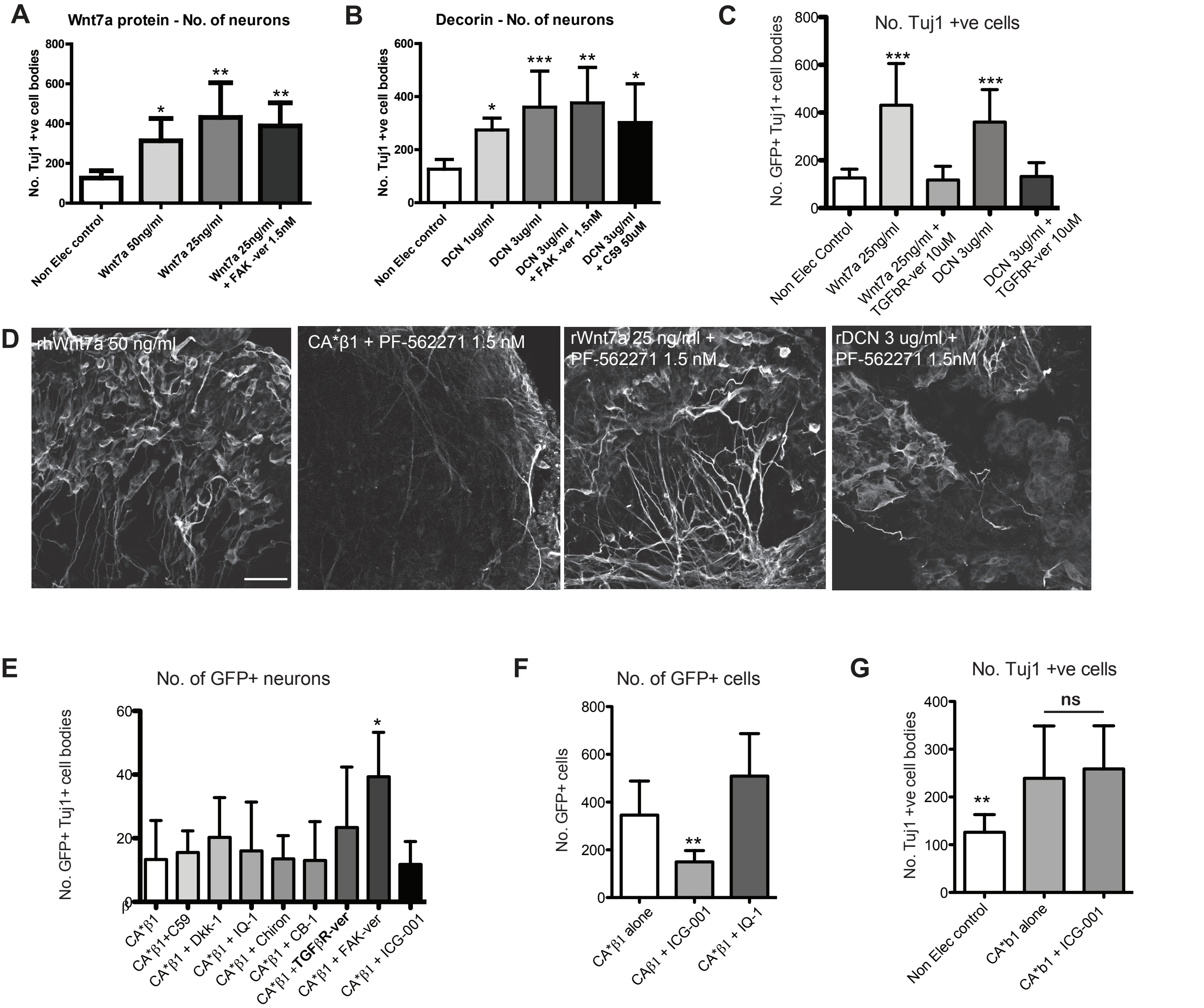

Supplementary Figure 8: Effect of perturbations of Wnt and FAK.

(A-C) Quantification of Tuj1+ cell bodies upon addition of recombinant Wnt7a and Decorin with addition of C-59,FAK inhibitor PF-562271 and TGFβR-ver LY2109761. (D) Images of Tuj1 staining in explants. Scale bar 50μm. (E) Quantification of GFP+ Tuj1+ neurons. (F) Quantification of GFP+ cells. (G) Quantification of Tuj1+ cells. *=p<0.05, **=p<0.01, ***=p<0.001, n>3, mean and SD, One way ANOVA.

Supplementary Table 1. Integrin constructs.

Itgβ1 construct	Description	Mutation	Function
hWTβ1	Wild type itgβ1	None	As the endogenous integrin, requires activation (inside-out signalling) and ligand to signal
ECβ1	Extra-cellular itgβ1	Lacks the intracellular domain	Unable to signal at all as unable to bind to intracellular downstream effectors
CA*β1	Constitutively active itgβ1	Point mutation in the intracellular salt bridge that normally stabilizes the integrin dimer in its bent/inactive conformation	Already in the active conformation, able to signal as soon as ligand is present
